# Comparison of two immunoassays for the measurement of serum HE4 for ovarian cancer

**DOI:** 10.1016/j.plabm.2021.e00235

**Published:** 2021-05-09

**Authors:** Chloe E. Barr, Garth Funston, Luke T.A. Mounce, Phillip W. Pemberton, Jonathon D. Howe, Emma J. Crosbie

**Affiliations:** a Division of Gynaecology, Manchester University NHS Foundation Trust, Manchester Academic Health Science Centre, Manchester, UK; b The Primary Care Unit, Department of Public Health and Primary Care, University of Cambridge, Cambridge, UK; c Institute of Health Research, University of Exeter Medical School, Exeter, UK; d Department of Clinical Biochemistry, Manchester University NHS Foundation Trust, Manchester Academic Health Science Centre, Manchester, UK; e Division of Cancer Sciences, University of Manchester, Faculty of Biology, Medicine and Health, Manchester, UK

**Keywords:** HE4, Biomarker, Immunoassay, Ovarian cancer detection

## Abstract

**Introduction:**

The use of Human Epididymis Protein 4 (HE4) as a biomarker for ovarian cancer is gaining traction, providing the impetus for development of a high throughput automated HE4 assay that is comparable to the conventional manual enzyme immunometric-assay (EIA). The aim of this study was to compare two immunoassay methods for the measurement of serum HE4.

**Materials and methods:**

1348 serum samples were analysed for serum HE4 using both the EIA and the automated chemiluminescent immunoassay (CLEIA) methods. HE4 values were compared using a Passing-Bablok regression and agreement assessed using Lin's concordance correlation coefficient (CCC). The absolute and percentage bias of the CLEIA compared to EIA was determined.

**Results:**

There was moderate agreement between the two methods (CCC 0.929, 95%CI 0.923-0.936). Passing-Bablok regression demonstrated an overestimation of the CLEIA [constant 4.44 (95%CI 2.96-5.68), slope 1.04 (95%CI 1.02-1.07)]. The CLEIA method had a mean percentage bias of 16.25% compared to the EIA method.

**Conclusion:**

The CLEIA significantly overestimated serum HE4 values compared to the EIA, which could impact clinical interpretation and patient management. Further studies are required to develop an appropriate cut-off depending on the population being investigated and the analytic method being used.

## Glossary

CA125: Cancer antigen 125
CCC: Lin's concordance correlation coefficient
CI: Confidence interval
CLEIA: Chemiluminescent immunoassay
EIA: Enzyme immunometric assay
FDA: Food and Drug Administration
GP: General Practitioner
HE4: Human epididymis protein 4
IU/L: International units per litre
MAb: Mono-clonal antibody
MFT: Manchester Foundation Trust
NICE: National Institute of Clinical Excellence
pmol/L: picomole per litre
ROMA: Risk of Malignancy Algorithm
UK: United Kingdom
USA: United States of America

## 1 Introduction

Ovarian cancer is the sixth most common cancer in women and the leading cause of death from gynaecological malignancy in the United Kingdom (UK) [1]. Cancer antigen 125 (CA125) is the most widely used diagnostic biomarker for ovarian cancer, however it lacks the diagnostic accuracy to reliably detect ovarian cancer at an early stage. There has been growing interest in human epididymis protein 4 (HE4) as an additional serum biomarker for ovarian cancer. HE4 has been approved by the United States Food and Drug Administration as a component of the Risk of Malignancy Algorithm (ROMA) for determining pre-surgical risk of ovarian cancer and monitoring of advanced disease[2].

With clinical use of HE4 expanding, a high throughput automated assay that allows multiple samples to be analysed rapidly is needed. The manual enzyme immunometric assay (EIA), developed by Fujirebio, was the first clinically-applicable HE4 assay. Fujirebio have since developed an automated analyser, Lumipulse®G, which uses a chemiluminescent immunoassay (CLEIA) technology, and calibrators that are traceable to the preparation used by the EIA (Recombinant human Fc antibody fragment- HE4 fusion protein) [3].

In meta-analyses reporting the pooled sensitivity and specificity of HE4 for ovarian cancer detection, there is marked heterogeneity in assay method and cut-offs applied[4,5]. Analytes measured by immunoassay show method-related differences in concentration, yet decision limits for biomarkers including CA125 are fixed across laboratories and guidelines[6]. It is important to compare new analytical methods with existing ones to ensure that results are comparable and are appropriately interpreted. This study aimed to compare serum HE4 levels measured by Lumipulse®G against the standard EIA, and to optimize assay-specific cut-offs for clinical use.

## 2 Materials and Methods

The study population included all primary care requested serum CA125 samples received by the Manchester University NHS Foundation Trust (MFT) Clinical Biochemistry laboratory between Apr-2018 and Apr-2019. Samples were collected from women who presented to their general practitioner (GP) with symptoms of possible ovarian cancer. Venous blood samples were collected using a serum separating vacuum tube containing silica particles and serum separating gel. Samples were centrifuged for 10min at 3000 rpm, tested for CA125 according to standard clinical protocols, and residual serum stored at -80 °C until analysis. The stability of frozen serum has been reported previously(3). Residual samples were tested for HE4 in a study investigating the utility of serum biomarkers for ovarian cancer risk assessment. The study received ethical approval from South Oxford B Research Ethics Committee and was registered prospectively on the ISRCTN clinical trial registry (ref:13470572).

Samples were tested for HE4 in weekly batches. On the day of testing, samples were thawed thoroughly to room temperature and mixed. Samples were analysed using the HE4 EIA (Fujirebio HE4 EIA, Oxford Biosystems, UK) and the Lumipulse® G600II analyser (Fujirebio, Gent, Belgium) in accordance with the manufacturer's instructions.

The EIA is a solid phase non-competitive immunoassay using mouse monoclonal antibodies (MAb) 2H5 and 3D8 directed against different HE4 epitopes. The maximum detection limit is 900 pmol/L and total coefficient of variation (CV) 3.6-6.5%. The EIA was performed on a 12x8 well microplate. Calibrators, controls and patient samples were tested in duplicate and a calibration curve constructed for each assay by plotting absorbance values versus concentration for each calibrator. HE4 concentration was determined from the calibration curve. Results were expressed as the mean of duplicate concentrations. The inter-assay CV was 8.9% and 5.9% for controls 1 and 2 respectively, and average CV of the duplicates was 4.1%.

The CLEIA uses a two-step sandwich immunoassay technique. The immunoreaction cartridges contain two monoclonal antibodies, MAb 2H5 and alkaline phosphatase (ALP)-labelled MAb 12A2. The maximum detection limit is 1500 pmol/L and the total CV is 3.4-5.5%. The analyser was calibrated every 28 days, when a new batch of immunoreaction cartridges was used or if the control was out of range. Controls were run before and after each batch of patient samples. Patient samples were run and tested in duplicate, and the mean concentration of duplicates recorded. HE4 concentration was calculated from a calibration curve. The total CV for controls 1 and 2 was 6.2% and 6.1% respectively and CV of the duplicates was 1.95%.

Data were analysed using a Passing-Bablok regression, which allows for investigation of systematic bias between the comparator (CLEIA) and standard (EIA) methods. Lin's concordance correlation coefficient (CCC) was used to estimate overall agreement between methods. The degree of absolute (CLEIA-EIA) and percentage (CLEIA-EIA)/EIA) bias of CLEIA values relative to EIA values were explored graphically using Bland-Altman plots[7]. Ordinary least squares regressions estimated the association of EIA-derived HE4 concentrations with absolute and percentage bias to determine whether degree of bias in CLEIA-derived values varied with HE4 concentration, with fractional polynomial models considered if the relationship was clearly non-linear[8]. Bias goals were identified from the HE4 biological variability in postmenopausal women; ‘desirable’ bias fell within ±4.7%, and ‘minimum quality’ bias within ±7.0% [9].

## 3 Results

**Fig. 1:**
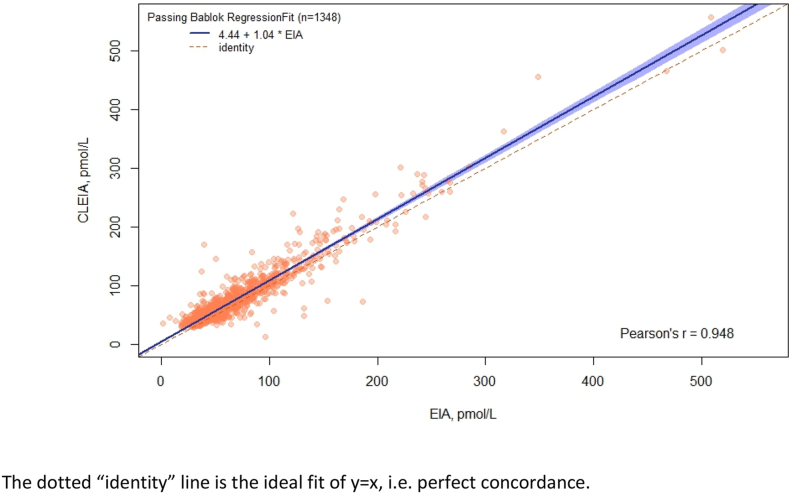
Passing-Bablok regression of the comparison of HE4 concentrations measured under CLEIA relative to EIA.

1348 GP requested serum CA125 samples were included in the study. Passing-Bablok regression (Fig. 1) showed that CLEIA systematically overestimated concentrations relative to EIA, with a constant of 4.44 (95%CI 2.96-5.68) and a slope of 1.04 (95%CI 1.02-1.07).

The dotted “identity” line is the ideal fit of y = x, i.e. perfect concordance.

Lin's CCC was 0.929 (95%CI 0.923-0.936), with a Pearson's correlation coefficient of r = 0.948. These indicate only moderate concordance overall, mostly due to a lack of precision (Pearson's correlation) rather than accuracy, which cannot be resolved by calibration[10]. Summaries of the absolute and percentage bias of CLEIA relative to EIA are shown in Table 1, together with the regression equation for the association of EIA-derived HE4 concentrations with each bias.

**Table 1:** Absolute and percentage bias of CLEIA relative to EIA, and regression equations estimating the association of EIA-derived concentrations with each bias.

Bias	Mean	SD	95% CI	Regression equation
Absolute	8.29 pmol/L	15.02 pmol/L	7.48–9.09 pmol/L	Bias = 6.84 pmol/L (95%CI 4.61 to 9.08) + 0.02 (95%CI -0.02 to 0.06) EIA
Percentage^a^	16.25%	25.67%	14.88%–17.62%	Bias^b^ = -2.19% (95%CI -4.01 to -0.36) + 986.83 (95%CI 659.16 to 1314.50) EIA^(-2) + 11442.99 (95%CI 10506.72 to 12379.27) EIA^(-2)*ln(EIA)

a Due to very large percentage differences at very low EIA HE4-concentrations, two outliers were excluded (#1; EIA = 1.4, CLEIA = 35.9: #2; EIA = 7.8, CLEIA = 46).

b Regression equation is from a fractional polynomial regression with exponents (-2, -2).

**Fig. 2:**
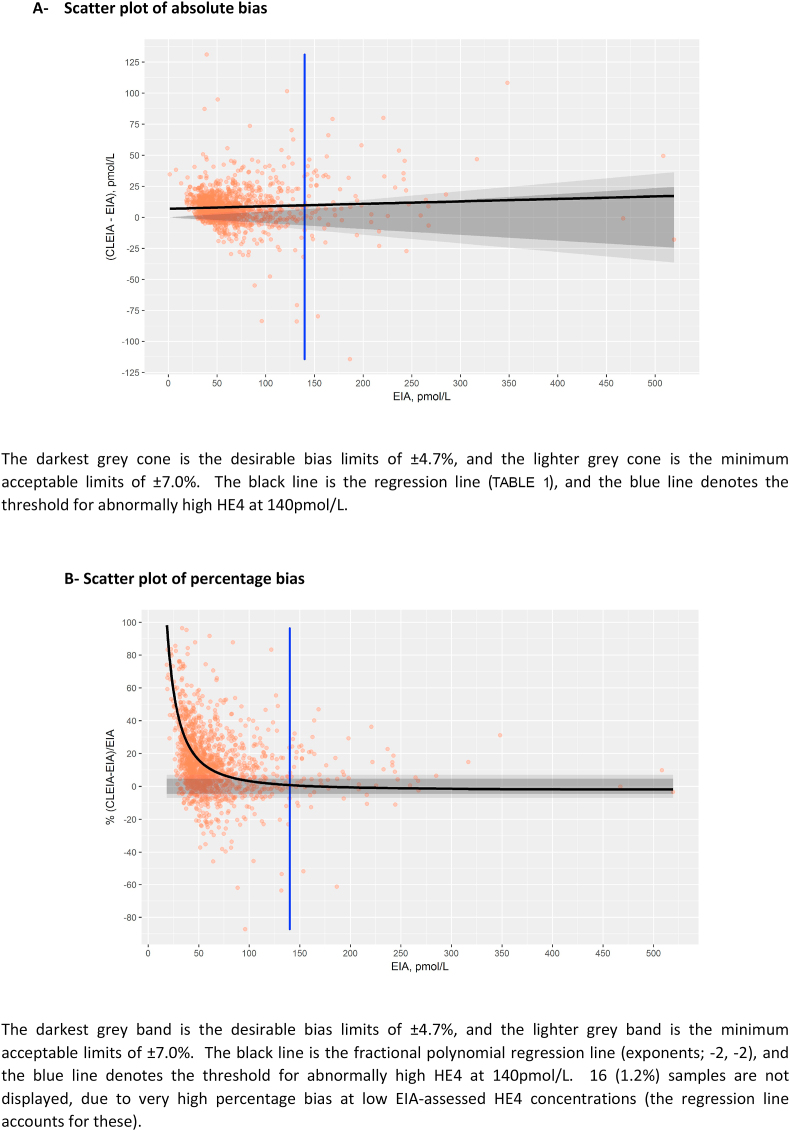
Scatter plots of bias.

Fig. 2 demonstrates the absolute and percentage bias. Absolute bias was outside the minimum quality bias level (7%) at HE4-concentrations below 140 pmol/L, but was within this bias limit over this value on average (Fig. 2A). Percentage bias tended to be high for lower HE4 concentrations, but fell within bias limits before the clinical threshold of 70 pmol/L (Fig. 2B).

Using the Passing-Bablok regression equation, an equivalent threshold for two clinically used thresholds for an abnormally high HE4 (>140 pmol/L and >70 pmol by EIA) would be 150 pmol/L and 77.2 pmol/L for the CLEIA, respectively.

## 4 Discussion

We have demonstrated a significant difference in serum HE4 values measured using CLEIA and conventional EIA. There was moderate agreement between the two methods, but CLEIA significantly overestimated HE4 values when compared to EIA, with a mean percentage bias of 16.25%. Identified biases of 7.2 pmol/L and 10 pmol/L, at the thresholds of 70 pmol/L and 140 pmol respectively, are likely to significantly impact clinical interpretation and decision making when using values measured by the CLEIA, highlighting the importance of assay-specific cut-offs for clinical application. The CLEIA manufacturer recommends cut-offs of 90 pmol/l for pre-menopausal women and 135 pmol/l for post-menopausal women, but few studies have compared different HE4 assays for clinical use. Using a much smaller sample size of 115 patients, only one previous study compared the CLEIA (using the Fujirebio Lumipulse®G) to the manual EIA, concluding that the CLEIA overestimated serum HE4 values across the range of measurements, with a 21% positive bias at a 140 pmol/L cut-off(3). Our study validates these findings and establishes the need for CLEIA-specific cut-offs for clinical application.

Our findings may be explained by the fact that the CLEIA and EIA HE4 assays use a different detection antibody; MAb12A2 and MAb3D8, respectively. MAb3D8 recognises epitopes expressed on the C-terminal domain but with a much lower binding affinity than MAb12A2, which binds to the N-terminal domain[11]. This suggests that differences in HE4 values measured by the two immunoassays may reflect differences in both HE4 variant detection and antibody binding affinity.

This study measured performance of the two immunoassays in a large population and reflects the experience of a working clinical laboratory, incorporating reagents from different batches. Whilst this is a strength in terms of clinical interpretation, it may be a limitation for accurate methods comparison. The samples collected were mainly concentrations <140 pmol/L, therefore assessment of concordance is less reliable at higher levels due to greater variation. Different technicians performed the CLEIA (CB) and EIA (PP, JH) analyses, which may have influenced concordance of the results. A lack of clinical outcome data hinders interpretation of our results in the context of its intended use, that is, as an early detection biomarker for ovarian cancer.

With increasing clinical utility of serum HE4 it is vital that those who use it consider analytical performance of the assay method used to quantify it. HE4 levels are impacted by age and most studies suggest a cut-off of 70 pmol/L for pre-menopausal and 140 pmol/L for post-menopausal women[12]. This study shows that equivalent cut-offs for the CLEIA are 77.2 pmol/L and 150 pmol/L, respectively. This is an important consideration because overestimation of HE4 levels from the CLEIA method could result in unnecessary referrals to gynaecology clinic, further investigation and even surgery for some patients. From our cohort, this would affect as many as 7% of women at a threshold of 70 pmol/L and 1% at a threshold of 140 pmol/L, causing psychological and physical harm to patients, wasting money and diverting resources away from those who need them. Where serial HE4 measurements are used to monitor patients on cancer treatment, change in assay method used by a clinical laboratory or a move between hospitals by a patient could lead to misinterpretation of serum results and alterations to patient management.

In conclusion, we found that the CLEIA significantly overestimates HE4 values compared to the EIA, suggesting that a common clinical decision limit may not be appropriate. Further studies are required to develop an appropriate cut-off depending on the population being investigated and the analytic method being used.

## Author statement

EJC was Principal Investigator for the study and is its guarantor. GF and EJC designed the study and supervised its execution. CEB performed the CLEIA assays. PWP and JH performed the EIA assays. LM performed statistical analyses. CEB and EJC wrote the manuscript. All authors provided critical comment, edited the manuscript, and approved its final version.

## Funding statement

Lumipulse®G HE4 immunoassay kits were generously donated by Fujirebio for use in this research. Fujirebio played no other role in the conduct of this study, analysis of the data or the decision to publish. CEB was supported through an MFT Clinical Research Fellowship and EJC through the NIHR Manchester Biomedical Research Centre (IS-BRC-1215-20007). This work was supported by Wellbeing of Women (Award Reference ELS701). This article presents independent research funded by the 10.13039/100012411NIHR. The views expressed are those of the authors and not necessarily those of Fujirebio, the NHS, NIHR, or the Department of Health.

## Declaration of competing interest

The authors declare that they have no conflicts of interests.
